# Pharmacological Studies on the Antidiabetic, Antioxidant, and Antimicrobial Efficacies of *Commiphora myrrha* Resin in Streptozotocin-Induced Diabetes in Rats: A Preclinical Study

**DOI:** 10.1155/2023/5478267

**Published:** 2023-02-14

**Authors:** Rasha A. Mansouri, Aftab Ahmad, Roushdy M. M., Huda F. Alshaibi, Mahmoud Ragab

**Affiliations:** ^1^Department of Biochemistry, Faculty of Sciences, King Abdulaziz University, Jeddah 21589, Saudi Arabia; ^2^Health Information Technology Department, Faculty of Applied Studies, King Abdulaziz University, Jeddah, Saudi Arabia; ^3^Pharmacovigilance and Medication Safety Unit, Center of Research Excellence for Drug Research and Pharmaceutical Industries, King Abdulaziz University, Jeddah, Saudi Arabia; ^4^Department of Biology, College of Sciences and Arts at Khulis, University of Jeddah, Jeddah, Saudi Arabia; ^5^Botany and Microbiology Department, Faculty of Science, Al-Azhar University, Naser City, 11884 Cairo, Egypt; ^6^Information Technology Department, Faculty of Computing and Information Technology, King Abdulaziz University, Jeddah 21589, Saudi Arabia; ^7^Center of Artificial Intelligence for Precision Medicines, King Abdulaziz University, Jeddah 21589, Saudi Arabia; ^8^Department of Mathematics, Faculty of Science, Al-Azhar University, Naser City, 11884 Cairo, Egypt

## Abstract

**Results:**

The aqueous extracts of MAE were phytochemically analyzed, and the results revealed the presence of high concentrations of tannins, sterols, and isoprenoids (terpenoids), while steroids and flavonoids were found in moderate concentrations. The plant extract showed promising inhibition of the growth of gram-positive and gram-negative pathogens. It also showed that MAE has potential antihyperglycemic and antioxidant activities. Microscopic examination of the pancreas showed degenerative changes and atrophy associated with dilatation of the exocrine ducts in the STZ-induced diabetic rats, while the treatment revealed that the Langerhans islets were close to normal without any histopathological alteration.

**Conclusion:**

The present results suggested that an aqueous extract of MAE could be considered an efficient antidiabetic, antioxidant, and antimicrobial treatment in the future.

## 1. Introduction

Diabetes mellitus (DM) is a chronic metabolic disorder, which is characterized by a deficiency of insulin secretion by the pancreas and or insulin resistance in peripheral tissues. Both of these conditions lead to the accumulation of glucose in the blood commonly referred to as hyperglycemia. Long-term hyperglycemia is an identifying parameter for diabetes, and this increased level for a long time creates a severe injury to multiple organ systems of the body. In long-term hyperglycemic conditions, the excessive glucose in the blood reacts with hemoglobin and forms glycosylated hemoglobin (HbA1C). Accordingly, HbA1C measurement is directly related to blood glucose concentration and is considered a very sensitive index for glycemic control [1]. Furthermore, DM can cause glycation of body proteins, leading to secondary complications that might primarily affect various vital organs, mainly the kidneys, eyes, nerves, and blood vessels. Uncontrolled diabetes may lead to blindness, heart attacks, renal failure, stroke, and sometimes amputation of limbs [2]. In addition, DM is shown to be accompanied by an increment in oxidative stress [3, 4]. Malondialdehyde (MDA) is known as one of the common oxidative stress primary biomarkers, and high levels of MDA were in different tissues, including serum and plasma of patients suffering from diabetes [5]. Hence, it is of utmost importance to control the hyperglycemia to prevent the secondary complications of diabetes. The existing antidiabetic medications have numerous undesirable side effects; therefore, there is an increasing demand for natural antidiabetic products by diabetic patients to avoid the side effects of available marketed drugs [6]. The prevalence of DM is continuously rising, especially in developing countries and undeveloped and low-income countries. The International Diabetes Federation (IDF) reported that the global prevalence of diabetes is continuously rising. It is a big global challenge for the well-being of peoples and to make a healthy society. This is reported in the IDF that currently, about 537 million adults are suffering from diabetes mellitus. These figures are further predicted to increase to 643 million by the year 2030 and again to 783 million by the year 2045 [7]. Lifestyle change is a critical feature of diabetes care. To control type 2 DM, the consumption of healthy food, increase physical work and exercise, control of body weight, psychosocial care, and stopping tobacco use are important steps [8]. Medical plants have been reported to possess antidiabetic efficacy by different mechanisms of action like increasing insulin secretion by stimulating *β* cells, decreasing insulin resistance, preventing reabsorption of renal glucose, and rejuvenation of beta cells of pancreas in terms of size and numbers [9]. Several studies have been reported the antihyperglycemic efficacy of the medicinal plants. The hypoglycemic effects of these medicinal plants are helpful to rectify the metabolic abnormalities and also delay the progress of diabetes-associated complications [9]. As per the World Health Organization (WHO) guidelines, it is essential to prevent the diabetes and its associated complications to attain the better life. There have been strong emphases in the search of medicinal plants with antidiabetic potential. Natural products of plant origin might be promising lead candidates in the discovery of drug development with antidiabetic potential. Myrrh resin is one of the naturally derived substances obtained from the bark of the *Commiphora myrrha*. *Commiphora myrrha* is a tree of the Commiphora genus, which belongs to the Burseraceae family. Many species of the genus Commiphora are primary source of the production of oleo-gum resin known as myrrh. The word “myrrh” was derived from the Arabic word *murr*, meaning bitter [10]. Myrrh has unique medicinal properties: it can act as a carminative, anti-inflammatory, astringent, analgesic, antiseptic, diuretic, emmenagogue, and expectorant. Unani physicians traditionally used myrrh for the treatment of different illnesses, including inflammation, asthma, cough and cold, cancer, ulcers, indigestion, spasms, respiratory disorders, congestion of lungs, arthritic pain, wounds, leprosy, and syphilis. It is also used as a stimulant. It is traditionally used to increase menstrual flow and for the management of various gynecological problems including leucorrhea, menorrhagia, and amenorrhea. It is also found to be beneficial in the cervical stenosis and pelvic inflammatory diseases. This is also used as abortifacient and galactagogue [11]. Although myrrh has extensive uses in traditional medicine, few studies have examined its effects on the pancreas and its potential antidiabetic properties. Some medicinal plants may show antioxidant activities by reducing the reactive oxygen species (ROS) that may appear due to free radicals in the pancreas [12]. The phenolic compounds have a major role to play in protecting living tissues from the severe effects of ROS that are considered to be an important risk factor for causing acute cell damage [13]. Various studies have mentioned that reinforcing the antioxidant system could decrease the harmful effects of diabetes [14, 15]. Streptozotocin (STZ) is a broad-spectrum antibiotic. This is toxic to beta cells of pancreas, which causes destruction of these *β*-cells of pancreas in the mammals. STZ is widely used in the medical research to induce experimental diabetes in animals. The induction mechanism includes the generation of oxygen free radicals by STZ, which consequently damages the pancreas and destruction of *β*-cells. The pathological conditions of STZ-induced diabetes are identical to the type 2 DM in humans [16]. Therefore, the current study is aimed at examining the protective efficacy of aqueous extract of *C. myrrha* resin (MAE) on STZ-induced diabetes in the pancreas of female Sprague Dawley rats, in addition to its antioxidant and antimicrobial activities.

## 2. Material and Methods

### 2.1. Plant Resin Collection and Identification

Oleo-gum resins of *C. myrrha* (used as an aromatic plant and in traditional medicine in Saudi Arabia) were collected (August 2019) from a wild tree growing in Wadi Noeman at Makkah, Saudi Arabia (21°21′55.98^″^N and 40°11′27.03^″^E). Prof. M. Fadl (professor of plant taxonomy at Taif University, Saudi Arabia) identified the tree. The collected samples were deposited in the herbarium of the Biology Department at Taif University, and the ID number for the voucher specimen is Wadi Noeman, 2019, 10512 (TUH)-Roushdy M.M. The criteria for choosing the best and ideal form of oleo-gum resin of *C. myrrha* included important characters such as its transparency, color, odor, and time of storage. The gum should not be stored for more than three months and should be transparent with a golden to brownish yellow color.

### 2.2. *C. myrrha* Resin Aqueous Extraction (MAE)

The dried powdered resin (100 g) of *C. myrrha* was washed with distilled water and left to dry at 60°C overnight. It was then subjected to extraction with 500 mL tap water at room temperature for 48 hours. The extraction step was followed by a filtration process using a Whatman No. 1 paper. The filtrates were concentrated using a rotary evaporator under reduced pressure and controlled temperature, followed by room temperature drying. A stock solution was prepared by dissolving the dried extract powder (400 mg) in distilled water (1000 mL) and stored in the refrigerator at 2-4°C for further investigations.

### 2.3. Preliminary Phytochemical Study

To identify the chemical constituents of the plant extract, the MAE powder was dissolved in distilled water and subjected to preliminary phytochemical screening. The aqueous extracts of *C. myrrha* were subjected to preliminary phytochemical investigations to determine the different phytoconstituents like terpenoids, sterols, and tannins, while steroids and flavonoids using standard official procedures [17, 18].

### 2.4. Experimental Animals

A total of 30 adult female albino Sprague Dawley rats of similar age and weight (120-140 g), obtained from the Egyptian Organization for Biological Products and Vaccines (Helwan, Cairo, Egypt), were used in the present study. For a 48-hour adaptation period, all rats were housed in individual stainless-steel cages (three rats/cage) and maintained at appropriate temperature (23 ± 2°C) and humidity (55% ± 10) with a standard 12 h light/dark cycle and ad libitum access to water and standard food. Body weights of all experimental rats were recorded weekly throughout the feeding period, and the body weight gain was calculated at the end of the experiment. The experimental animals were handled in compliance with the principles of good laboratory practices and ethical guidelines on animal use in research, and the study protocol was approved by the research ethics committee (REC/NHTMRI/A5-2021).

### 2.5. Induction of Diabetes

DM was induced in the rats that had fasted overnight by one intraperitoneal injection (60 mg/kg body weight) of STZ (Sigma Chemicals Co., St. Louis, USA) that was prepared in a fresh and cold sodium citrate buffer (0.1 M citric acid and 0.1 M trisodium citrate dihydrate) at pH 4.5 [19]. Blood samples were collected by tail snip method, and the sugar level of each animal was measured before the treatment (day 0) and 72 h post-STZ treatment. Rats were considered to be hyperglycemic based on blood glucose levels > 200 mg/dL [20].

### 2.6. Study Design

The thirty rats were randomly allocated into three main groups (*n* = 10 per group) as required by the present study. Animals were randomly allocated into three groups as follows:

1. Group I (normal control group): the rats were given intraperitoneal injection of sodium citrate buffer solution and marked as NCG. The rats were fed on a standard diet and left without treatment under the same laboratory condition
2. Group II (diabetic control group): the rats were given with a single intraperitoneal injection of 60 mg/kg, of body weight of STZ, and marked as DCG. They were also fed a standard diet under the same laboratory condition
3. Group III (MAE-treated group): the rats were first injected with STZ (60 mg/kg body weight) for the induction of diabetes and marked as aqueous extract of *Commiphora myrrha* resin-treated group (MAETG). The diabetic rats were treated with MAE powder at 0.5 mL of 0.5 g/kg body weight dissolved in distilled water. The treatment was given for 30 days. The treatment with MAE was performed orally by gastric intubation between 9:00 am and 11:00 a.m. for 30 days. The body weight was measured at the beginning and the end of the experiment

### 2.7. Sample Collection

At the end of the experimental period (30 days), the rats were fasted overnight and anesthetized with urethane (99%, Aldrich) at a dose of 1 g/kg body weight intraperitoneally. Blood samples were taken from the retroorbital venous plexus. The samples were allowed to coagulate at room temperature and centrifuged at 4000 revolutions per minute (RPM) for 15 minutes until the serum was separated and stored at −20°C for further biochemical investigations.

### 2.8. The Antimicrobial Activities of MAE

#### 2.8.1. Microorganisms

The antimicrobial activities of MAE were evaluated against various pathogenic bacterial strains including both gram-positive and gram-negative bacteria. The strains used for the antimicrobial assays were obtained from American Type Culture Collection (ATCC, Rockville, MD, USA). Gram-negative strains were *Salmonella typhimurium* (ATCC 13311), *Escherichia coli* (ATCC 10536), *Pseudomonas aeruginosa* (ATCC 27853), *Pseudomonas fluorescens* (ATCC 13525), and *Klebsiella pneumoniae* (ATCC 10031), while the gram-positive bacteria comprised *Bacillus subtilis* (ATCC 11774), *Streptococcus pyogenes* (ATCC 12344), and *Staphylococcus epidermidis* (ATCC 12228). Bacterial cells were cultivated on Mueller-Hinton agar medium at pH 7.4. The agar plates were incubated at 37°C for 24 h.

### 2.9. Antibacterial Assay Using Agar Disc Diffusion Method

The antibacterial activity of MAE was carried out using the agar disc diffusion method [21]. Each bacterial strain was first cultivated in nutrient broth at 37°C for 24 h. Each bacterial suspension was diluted with nutrient broth to obtain inocula of ~1 × 10^6^ CFU/mL [22]. One milliliter of the standardized inoculum of each test bacterium was spread with the help of a sterile spreader onto a sterile nutrient agar plate. The plates were allowed to dry. A Whatman No. 1 sterile filter paper disc (6 mm diameter) was impregnated with 100 *μ*L of 10 mg/mL of the aqueous solution of myrrh resin. The preparation of negative controls was carried out using sterilized distilled water. Subsequently, the plates were refrigerated for at least 1 h for diffusion to take place and then incubated at 37°C for 24 h. Evaluation of antibacterial activity was determined by measuring the resulting inhibition zones' diameter against the tested bacteria. Three replicates of the experiment were carried out, and the zone of inhibition was measured in millimeters [23]. One hundred microliters of ciprofloxacin was loaded onto filter papers and used as a positive control.

### 2.10. Biochemical Analysis

Biochemical tests, including fasting plasma glucose, total cholesterol (TC), triglycerides (TG), high-density lipoprotein (HDL-C) cholesterol, HbA1C, alkaline phosphatase (ALP), alanine aminotransferase (ALT), aspartate aminotransferase (AST), gamma-glutamyl transferase (GGT), and total and direct bilirubin (T and DB) were measured in a Roche Cobas 6000/c501 chemistry automated analyzer device (Roche Diagnostics, Mannheim, Germany) using the Roche laboratory kit reagent according to the reference range of the Cobas c501 biochemistry analyzer. Fasting insulin was measured in the Roche Cobas 6000/e601 hormone automated analyzer device (Roche Diagnostics, Mannheim, Germany) using the Roche laboratory kit reagent according to the reference range of the Cobas e601 hormone analyzer. The homeostasis model assessment of insulin resistance (HOMA IR) was calculated as the product of the fasting serum glucose (mg/dL) and fasting insulin levels (mU/L) divided by a constant (405) according to the formula of Pickavance et al. [24]. Serum levels of total antioxidant capacity (TAC), as well as MDA, were assessed by the enzyme-linked immunosorbent assay (ELISA) method (Spectrum, Egypt) according to the method of Miller et al. [25] and Jiang et al. [26], respectively. Low-density lipoprotein (LDL-C) cholesterol levels were calculated according to the formula of Mousavi et al. [27]:

(1) $$\begin{array}{c} \text{LDL}‐\text{C}=\text{Cholesterol}–\left(\text{HDL}‐\text{C}+\frac{\text{TG}}{5}\right). \end{array}$$

### 2.11. Pancreatic Harvesting and Tissue Homogenate Preparation

At the end of 30 days, all the rats were anesthetized with urethane (99%, Aldrich) at a dose of 1 g/kg body weight intraperitoneally, and the pancreas of each rat was quickly removed and washed in ice-cold saline immediately. The harvested pancreas was divided into two halves: one-half was saved for the histopathological examination, and the other half was used to prepare the pancreatic homogenate for biochemical analysis. The pancreatic homogenate was prepared in 10 mL of ice-cold phosphate-buffered saline (PBS) using a mechanical homogenizer. Samples were then centrifuged at 1000 × g for 10 min at 4°C to remove large insoluble particles. Finally, the supernatant was separated and stored at -80°C in aliquots for further biochemical analysis and measurement of MDA levels [26].

### 2.12. The Histopathological Analysis of Pancreatic Tissue

The harvested pancreas from the tested animals was fixed in 10% formalin and embedded in paraffin. Paraffin-embedded tissue blocks were prepared, and 5 *μ*m thick sections were taken using a microtome for further staining. Briefly, the sections for histopathological examination were placed on glass slides, deparaffinized, rehydrated, and stained with routine hematoxylin and eosin (H&E) stain. The stained slides were covered with coverslips after mounting and examined under a light microscope [28].

### 2.13. Statistical Analysis

All data were expressed as mean ± SD (standard deviation). Analysis of variance (ANOVA) was done, followed by the post hoc least significant difference test (LSD) to test the research hypothesis. Data analyses were performed using the statistical package for social sciences (SPSS version 26) (IBM Corp., Armonk, N.Y., USA). The differences between the groups were considered statistically significant if *p* value was <0.05.

## 3. Results

### 3.1. The Phytochemical Screening of MAE

The results of the phytochemical screening of MAE revealed the presence of high concentrations of terpenoids, sterols, and tannins, while steroids and flavonoids were found in moderate concentration (Table 1).

### 3.2. Effect of MAE on Bacterial Activity

MAE showed promising inhibition of the growth of the tested pathogens, as shown in Table 2. The maximum inhibition zones were found against *E. coli* (ATCC 10536) followed by *S. epidermidis* (ATCC 12228), *B. subtilis* (ATCC 11774) (27 mm), *S. pyogenes* (ATCC 12344), *K. pneumoniae* (ATCC 10031), *P. aeruginosa* (ATCC 27853), *P. fluorescens* (ATCC 13525), and *S. typhimurium* (ATCC 13311), respectively. The extract showed high activity against all the tested strains, when compared to the referenced antibiotic (ciprofloxacin).

### 3.3. Effect of MAE on Body Weight, Food Intake, and Water Intake Changes

The effect of MAE on body weight, food intake, and water intake in the diabetic control group (DCG) and MAETG is observed in Table 3. The results showed that there were no significant differences between groups in body weight, food intake, and water intake at the beginning of the experiment.

However, by the end of the study, the STZ-induced DG exhibited a greater loss of body weight in comparison to NCG. In contrast, the body weight of MAETG was shown to be significantly increased compared with DG and significantly decreased compared to NCG (*p* < 0.001). The opposite changes were seen with food and water intake. Therefore, MAE could induce weight loss but increase food and water intake (*p* < 0.003).

### 3.4. Effect of MAE on Liver Function

**Figure 1:**
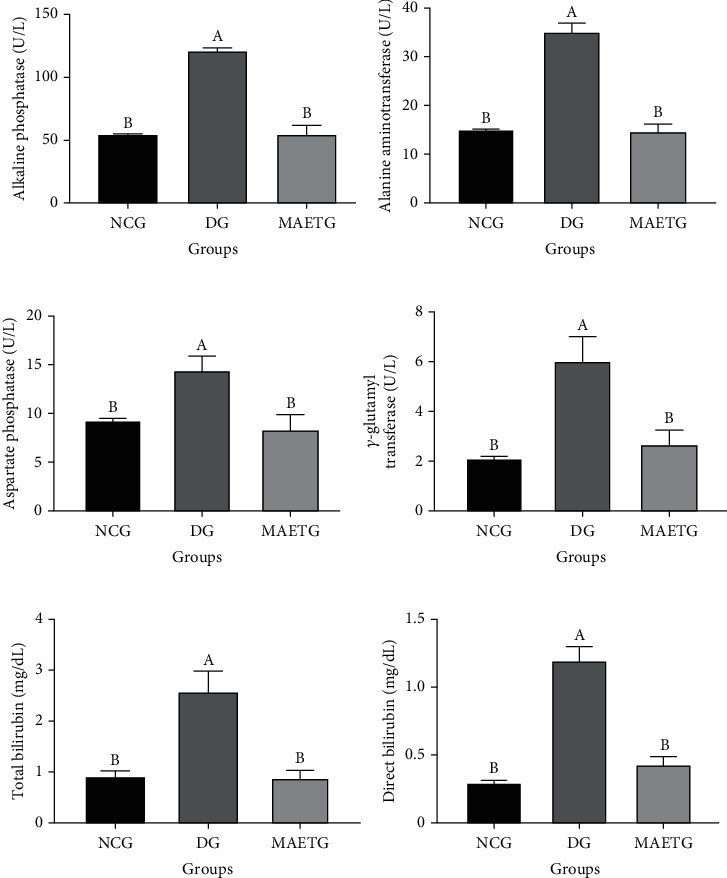
The effects of MAE of *C. myrrha* on liver function parameters ((a) alkaline phosphatase, (b) alanine aminotransferase, (c) aspartate phosphatase, (d) gamma-glutamyl transferase, (e) total bilirubin, and (f) direct bilirubin) of STZ-induced diabetic rats. Data were presented as mean ± SD. Data were analyzed using ANOVA followed by LSD. The mean difference is significant at *p* < 0.05. Each group contained 10 rats. Different superscript letters (A and B) denote significance, while similar letters denote no significance between groups.

Serum ALP (*p* < 0.0012), ALT (*p* < 0.001), AST (*p* < 0.0316), GGT (*p* < 0.001), TB (*p* < 0.001), and DB (*p* < 0.001) levels in STZ-induced diabetic rats (DG) were significantly elevated when compared to the NCG and MAETG (Figure 1).

### 3.5. Effect of MAE on Serum Lipid Profile

**Figure 2:**
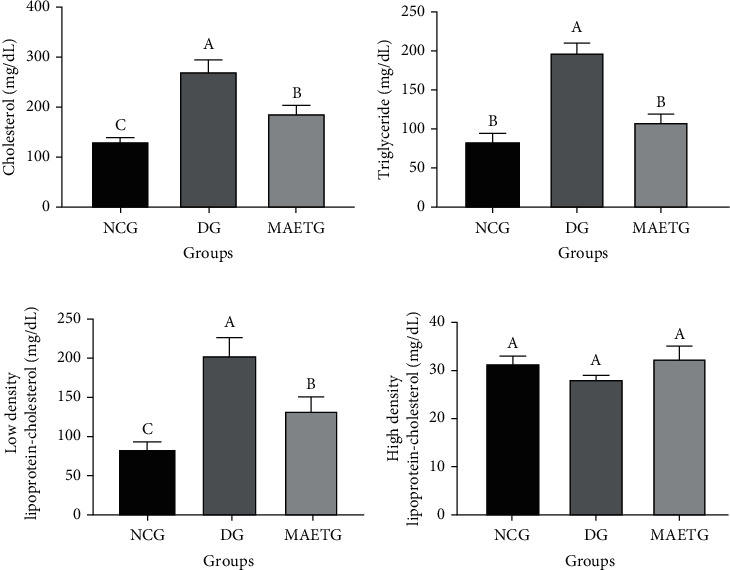
Effect of MAE on (a) total cholesterol, (b) triglyceride, (c) low-density lipoprotein cholesterol, and (d) high-density lipoprotein cholesterol in STZ-induced diabetic rats after 30 days of treatment. Data were presented as mean ± SD. Data were analyzed using ANOVA followed by LSD. The mean difference is significant at *p* < 0.05. Different superscript letters (A, B, and C) denote significance while similar letters denote no significance between groups. Each group contained 10 rats.

Although there were no significant changes in HDL-C values between the groups (*p* < 0.864), DG exhibited significant elevation in serum levels of TC (*p* < 0.001), TG (*p* < 0.001), and LDL-C (*p* < 0.001) when compared to NCG and MAETG. In contrast, MAETG showed an obvious reduction in the serum levels of these parameters when compared to DG, even though they were still higher than the normal group (Figure 2).

### 3.6. Effect of MAE on Fasting Blood Glucose and Insulin Levels, HOMA-IR, and HbA1C

As shown in Table 4, STZ injection was shown to significantly increase the levels of the fasting serum glucose (*p* < 0.001), HbA1C (*p* < 0.001), and HOMA-IR (*p* < 0.001) in DG when compared to NCG and MAETG, and it caused a significant decrease in the fasting serum insulin (*p* < 0.005) within DG.

### 3.7. Effect of MAE on Antioxidant Activity

**Figure 3:**
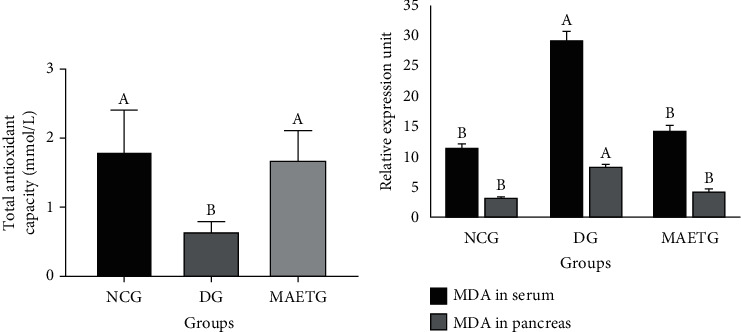
Effect of MAE on (a) TAC (total antioxidant capacity) and (b) MDA (malondialdehyde) in STZ-induced diabetic rats after 30 days of treatment. Data were presented as mean ± SD. Data were analyzed using ANOVA followed by LSD. The mean difference is significant at *p* < 0.05. Different superscript letters (A and B) denote significance while similar letters denote no significance between groups. Each group contained 10 rats.

Correspondingly, the intraperitoneal injection of STZ in albino rats showed an imbalance in the oxidative status which was confirmed by a significant reduction in serum TAC (*p* < 0.008) and a significant elevation in serum MDA (*p* < 0.005) compared to NCG and MAETG (Figure 3). The results showed that MAETG improved the levels of serum TAC and MDA when compared to the DG rats.

Similarly, MDA in the pancreatic tissue of diabetic rats (DG) was significantly increased (*p* < 0.001) in comparison with NCG and MAETG.

### 3.8. Effect of MAE on the Histological Structure of the Pancreas

**Figure 4:**
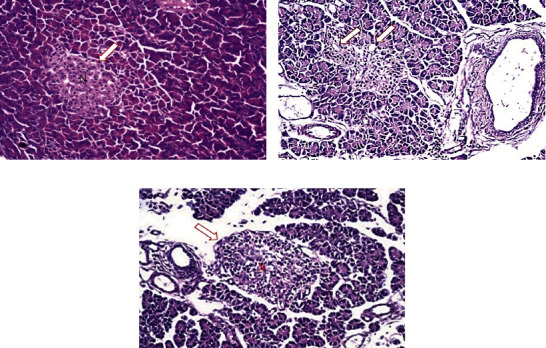
Effect of MAE of *C. myrrha* on pancreatic Langerhans islet cells after 30 days in albino rats: (a) histological appearance of Langerhans islets of normal control rats (NCG) showing no histopathological changes, (b) histological appearance of Langerhans islets of STZ-induced diabetic rats (DG) showing degenerative changes and atrophy associated with dilatation of the exocrine ducts (see arrow), and (c) histological appearance of Langerhans islets of the treated group (MAETG) showing the normal histopathological pattern.

The light microscopic observation of pancreatic islet cells from normal rats (NCG) showed no histopathological alteration, with a normal histological structure of the islet of Langerhans cells as endocrine portion as well as the acini and duct system of the exocrine portion (Figure 4(a)). However, the microscopic examination of DG showed degenerative changes and atrophy associated with dilatation of the exocrine ducts. This was a result of STZ action as a diabetic induction agent (Figure 4(b)). MAETG, on the other hand, showed marked improvement in the histological appearance of Langerhans islets with normal pattern (Figure 4(c)).

## 4. Discussion

Although there are different types of drugs available to lower blood glucose levels in humans, these medications are known to cause numerous adverse effects. Therefore, researchers are focusing on assessing other treatment options, including the search for active natural products.

Natural products have exhibited a range of biological properties, including anticancer, antioxidant, antimicrobial, and anti-inflammatory. *C. myrrha* has been investigated and reported to exhibit a wide range of therapeutic efficacies since the discovery of this medicinal plant [10]. Therefore, the present study was conducted to assess the antimicrobial and hypoglycemic potential activities of *C. myrrha* resin aqueous extract.

In DM patients, uncontrolled hyperglycemia conditions are among the most serious factors that may disrupt the immune system [29]. Another factor that may lead to type 2 DM is malnutrition (especially deficiency in vitamin D). Vitamin D deficiency has been reported to be associated with insulin resistance, type 2 diabetes, cancer, obesity, and cardiovascular diseases [30–32].

Our results revealed that MAE has strong potency as an antimicrobial agent compared to the antibiotic, ciprofloxacin. The obtained result is in agreement with Alqahtani et al. [10], who stated that *C. myrrha* extract showed strong antimicrobial activity against gram-positive bacteria like *Enterococcus faecalis and S. aureus*. The research study ascribed the reason for this antimicrobial action of the plant extract due to its high concentrations of active compounds like 2-acetoxy-furano-diene, furano-eudesma-1,3-diene, and 2-methoxyfuranodiene including some other phytochemical constituents. The present results are in agreement with several studies that have investigated the potential effects of exercise and a low-calorie vegetarian diet on oxidative stress and insulin resistance in type 2 diabetic patients [33]. On the other hand, another study by Jeevandran et al. [34] investigated the effect of ethanolic extracts of seeds of *Archidendron pauciflorum* (ESAP), where the ESAP did not exhibit an antihyperglycemic effect in diabetic rodents. As a result, it concluded that not all plants or even plant parts have effects on diabetes.

To induce DM, STZ was intraperitoneally injected in the experimental animals [35]. From the present observations, STZ-induced hyperglycemia is characterized by a high level of low serum insulin level, high glucose level, and high HbA1C level with an elevation of calculated HOMA-IR. These results were in accordance with different studies. Iftikhar et al. [36] showed that the administration of MAE (0.5 g/kg b.w.) for 30 days considerably reduced the hyperglycemic action of STZ.

Furthermore, Al-Romaiyan et al. [37] stated that the uses of aqueous *C. myrrha* extract (2 mg/mL) rapidly and reversibly increased the secretion of insulin at both stimulatory and substimulatory glucose levels in islets of the pancreas of humans and mice.

In addition, DM is a metabolic disorder with hyperglycemia. The uncontrolled elevated level of blood glucose causes serious complications in many vital organs like the kidney, pancreas, liver, and heart [4]. Lipid abnormalities have been found in diabetic patients. Diabetes is associated with dyslipidemia like elevated serum triglycerides, increased cholesterol, elevated LDL, and reduced HDL. These lipid abnormalities have been observed in nearly 40% of patients suffering from diabetes [38, 39]. In the current study, the results of the lipid profiles are at par with the previously reported studies. The administration of STZ significantly increased the serum concentration of cholesterol, TG, and LDL, while it decreased the HDL-C levels. Another study reported similar results which investigated the association between diabetes and lipid profile in the blood. This study illustrated an increase in the levels of serum triglyceride, cholesterol, and LDL-C that indicated the incidence of hyperlipidemia in the rats. The underlying mechanism of hyperlipidemia could be due to the hyperactivity of hormone-sensitive lipase, which causes the flow of fatty acids from triglycerides deposited in the adipocytes [40]. In contrast, the results from our study demonstrated that the administration of MAE to diabetic rats significantly improved the lipid profile. Therefore, one can conclude that MAE proved to have antidiabetic and antihyperlipidemic activities against STZ-induced DM. Our results are at par with Ojiako et al. [41] and Ota and Ulrih [42], who stated that the *Commiphora myrrha* contains numerous active phytoconstituents like alkaloids, flavonoids, glycosides, and terpenoids, which exhibited substantial antioxidant antidiabetic properties. The flavonoid component of the plant extract could be responsible for the antidiabetic activities. The flavonoids might be responsible for the regeneration and survival of pancreatic beta cells since flavonoids are potential alpha-amylase inhibitors [7]. The sodium glucose cotransporter-1(SGLT1) is an intestinal glucose transporter that facilitates the transport of glucose into the bloodstream. The tannins and polyphenolic components of the MAE might act by inhibiting the SGLT1, hence inhibiting glucose uptake from the rat's intestine [43].

Therefore, the antidiabetic efficacy of the MAE might be explained due to the presence of a high amount of polyphenolic compounds and flavonoids including alkaloids in the aqueous extract.

Plants with clinical applications play a major role in the control of plasma glucose through various mechanisms, one of which is the increase in the number of beta cells in the pancreas and the activation of their regeneration ability [10]. Induction of diabetes by STZ could severely damage the pancreatic beta cells and subsequently decrease serum insulin levels [44]. This fact was also confirmed by several studies that revealed that STZ directly causes substantial destruction of the pancreatic beta cells [45, 46]. So, treatment with MAE could increase the serum insulin level, and this could be explained by the presence of flavonoids, tannins, and steroids in the investigated extract. Therefore, the plant extract may have a high antioxidant ability that may support the protection of beta cells from harmful oxidative stress and other damaging factors [47]. Coskun et al. [48] reported that flavonoids could significantly decrease blood sugar levels and also exhibited protective effects on the beta cells from oxidative stress and preserve the integrity of beta cells of the pancreas.

MDA is a byproduct of lipid peroxidation and is commonly known as a marker of oxidative stress. The present study revealed a significant increase in both serum and pancreatic MDA and a significant decrease in total antioxidant capacity (TAC) in the STZ-induced diabetic rats (DG).

The present study also revealed that there was a significantly increased level of MDA both in serum and pancreatic tissues. There was a significant reduction in the total antioxidant capacity (TAC) in the STZ-induced diabetic rats (DG). Interestingly, the administration of MAE to the diabetic animals significantly decreased the MDA in serum and pancreatic tissues but exhibited significant antioxidant activity, where TAC was significantly increased.

Several previous studies on STZ-induced diabetes also reported similar results [49–51]. These studies showed that the level of MDA in the tissues of the pancreas was significantly elevated as compared to the normal control group [52–54]. Furthermore, Jagtap and Patil [55] and Abou Khalil et al. [56] reported that MDA value was increased in the plasma as well as the pancreatic tissue of diabetes-induced rats, while it was significantly decreased by the treatment of diabetic animals with plant extracts (family Apiaceae). These results could be explained as mentioned by Alqahtani et al. [10], who reported that *C. myrrha* has a great variation in furano-sesquiterpenoids, 2-methoxyfuranodiene and 2-acetoxyfuranodiene contents, possessing maximum antioxidant activity.

Pancreatic histopathological studies showed degenerative changes and atrophy associated with dilatation of the exocrine ducts in the STZ-diabetic rats (DG) [56]. On the other hand, normal control animals as well as MAE-treated animals showed normal pancreatic Langerhans islets. Similar results were obtained by Parasuraman et al. [9], who found vacuolar degeneration in islet cells in sections from the pancreas of diabetic rats with the atrophic islet.

The present study gives hope for using this plant extract in many applications of clinical importance. The plant extract could be used as a mouthwash due to its antiseptic and antimicrobial properties. It also could be used for wound dressing for the prevention of serious infections. Its antimicrobial ingredients could be extracted in the future and tested to be used as an antibiotic after studying its side effects.

## 5. Conclusion

Our experiments focused on the extraction, identification, and purification of the active constituents of MAE, having promising biological activities. According to the results from the current study, one can conclude that the administration of *C. myrrha aqueous* extract has the ability to reduce the plasma glucose level in STZ-induced diabetes in rodents. The MAE also had antimicrobial and antioxidant potential activities. The antihyperglycemic, antioxidant, and antimicrobial effects of MAE may be due to the presence of high amounts of various active constituents like polyphenolic components and flavonoids including alkaloids in the aqueous extract. In addition, the histological microscopic examination of MAETG pancreases revealed that the Langerhans islets turned out to be normal without any histopathological alteration despite the injection of animals with STZ, which has a destructive effect on the pancreatic cells. The outcome of these findings advocates that MAE might be pondered as an effective oral antidiabetic therapy, including additional antioxidant and antimicrobial agents in the future.

*C. myrrha* showed high antioxidant as well as hypoglycemic activities due to the presence of various important constituents and compounds inside the plant. Therefore, the future prospect of the present study is to use simple, viable, effective, and rapid methodologies which will be essential for the extraction of these active phytochemicals and to study their effects on humans to acquire more information about this promising plant.

## Figures and Tables

**Table 1:** Phytochemical screening of *C. myrrha* extract.

S. no.	Constituents	Level^∗^
1	Terpenoids	+++
2	Sterols	+++
3	Steroids	++
4	Tannins	+++
5	Flavonoids	++

^∗^: ++: moderate concentration; +++: high concentration.

**Table 2:** *In vitro* antibacterial activity of *C. myrrha* oleo-gum extract against tested bacteria.

Test species	Zone of inhibition (mm)^∗^	
	Resin extract	Ciprofloxacin
*S. typhimurium* (ATCC 13311)	18.33 ± 0.056^f^	15.67 ± 0.029^f^
*E. coli* (ATCC 10536)	28.33 ± 0.044^b^	21.00 ± 0.076^b^
*P. aeruginosa* (ATCC 27853)	22.33 ± 0.012^e^	17.00 ± 0.088^e^
*P. fluorescens* (ATCC 13525)	21.00 ± 0.015^e^	15.00 ± 0.009^f^
*K. pneumoniae* (ATCC 10031)	25.33 ± 0.035^d^	18.33 ± 0.053^d^
*B. subtilis* (ATCC 11774)	28.33 ± 0.048^b^	20.67 ± 0.039^c^
*S. pyogenes* (ATCC 12344)	26.67 ± 0.103^c^	21.33 ± 0.011^b^
*S. epidermidis* (ATCC 12228)	28.67 ± 0.099^a^	23.67 ± 0.047^a^

^∗^Concentration of extracts 10 mg/mL (100 *μ*g/disc). Inhibition zones were the mean of three replicates. The mean results were expressed as mean ± SD. Different superscript letters (a, b, and c) denote significance, while similar letters denote no significance between groups. The mean difference is significant at *p* < 0.05.

**Table 3:** Effects of treatment with MAE on body weight, food intake, and water intake in STZ-induced diabetic rats.

Groups^∗^	Body weight (g)			Food intake (g/day)			Water intake (mL/day)		
	Day 1	Day 15	Day 30	Day 1	Day 15	Day 30	Day 1	Day 15	Day 30
NCG	116.3 ± 8.52	136.5 ± 10.64	160.6 ± 8.41^a^	17 ± 0.34	19.8 ± 0.93	24.1 ± 0.97^b^	46.1 ± 2.13	51.6 ± 3.55	56.3 ± 1.43^b^
DCG	120.2 ± 7.27	107.1 ± 10.59	95.4 ± 9.25^b^	18.4 ± 0.91	22.6 ± 2.26	29.2 ± 2.98^a^	45.5 ± 2.99	54 ± 3.94	62.7 ± 4.16^a^
MAE	118.3 ± 8.06	123.5 ± 11.06	134.3 ± 8.94^a^	18.1 ± 0.49	20.6 ± 1.56	25.5 ± 1.50^b^	45.7 ± 2.26	52.3 ± 4.74	57.9 ± 2.89^b^

^∗^Each group contained 10 rats. The mean results were expressed as mean ± SD. Different superscript letters (a, b, and c) denote significance, while similar letters denote no significance between groups. The mean difference is significant at *p* < 0.05.

**Table 4:** Effect of MAE of *C. myrrha* on serum glucose, fasting insulin, HbA1C, and HOMA-IR index in STZ-induced diabetic rats after 30 days of treatment.

Groups^∗^	Fasting glucose (mg/dL)	HbA1C (%)	Fasting insulin (mU/L)	HOMA-IR
NCG	92.3 ± 7.7^b^	4.998 ± 0.311^b^	1.917 ± 0.13^a^	0.44 ± 0.04^b^
DG	437.9 ± 45.36^a^	8.754 ± 0.59^a^	0.885 ± 0.13^c^	0.96 ± 0.29^a^
MAETG	84.7 ± 10.55^b^	4.751 ± 0.59^c^	1.579 ± 0.12^b^	0.33 ± 0.04^b^

^∗^Each group contained 10 rats. The mean results were expressed as mean ± SD. Different superscript letters (a, b, and c) denote significance while similar letters denote no significance between groups. The mean difference is significant at *p* < 0.05. HbA1C: glycated hemoglobin; HOMA-IR: homeostasis model assessment of insulin resistance.

## Data Availability

All the data are included in the manuscript.
